# The effects of zinc oxide eugenol, calcium hydroxide, bioactive glass, resin and bioceramic canal sealers on post-operative pain: A randomized clinical trial

**DOI:** 10.6026/973206300222079

**Published:** 2026-04-30

**Authors:** Shriya Mitna, Santosh Kumar Singh, Yashwani Jain, Kapil Malviya, Sakshi Agrawal

**Affiliations:** 1 Department of Conservative Dentistry and Endodontics, People's College of Dental Sciences and Research Centre, People's University, Bhopal, Madhya Pradesh, India

**Keywords:** Bioactive glass sealer, post-operative pain, root canal treatment, endodontic sealers, visual analog scale (VAS)

## Abstract

Post-operative pain after root canal treatment is still a common clinical issue that can make patients less comfortable and less likely to accept endodontic therapy. Therefore, it is of interest to assess post-operative pain associated with five distinct root canal sealers- bioactive glass-based, bioceramic-based, calcium hydroxide-based, resin-based and zinc oxide eugenol-based-using single-rooted premolars exhibiting symptomatic apical periodontitis. Hence, a total of 135 patients were randomly assigned to five groups (n = 27) and pain was measured using the visual analog scale at different time points after surgery. Bioactive glass-based sealers had the lowest pain scores after surgery, while zinc oxide eugenol-based sealers had higher pain levels in the first few days after surgery. Over time, pain levels decreased slowly across all groups. Thus, bioactive sealers may help reduce pain immediately after root canal treatment.

## Background:

Root canal therapy is a common procedure that aims to remove infection in the root canal system and preserve natural teeth. Even through endodontic therapy works well most of the time; patients may feel pain after the procedure. Post-operative pain is characterized as discomfort that arises following the commencement or conclusion of root canal therapy in teeth that were previously asymptomatic or when pre-existing pain intensifies post-treatment. It remains one of the most common problems that can occur during endodontic procedures and it can make patients less likely to trust dental care and to agree to future treatment. Research indicates that post-operative pain following root canal treatment may manifest in approximately 2.5% to 60% of cases, typically peaking within the initial 24 hours post- treatment and subsequently diminishing over several days [1, 2- 3]. Post-operative pain is a multifactorial phenomenon influenced by various biological and procedural factors. These include pain before surgery, the condition of the pulp and periapical tissue, the tooth type, the instruments used, the irrigation protocols and the methods used to fill the tooth [4]. Mechanical irritation of periapical tissues, extrusion of infected debris, chemical irritation from irrigants or filling materials and host inflammatory responses may all contribute to post-operative discomfort [5, 6]. Furthermore, the materials employed in obturation may affect the inflammatory response in periapical tissues. Root canal sealers are very important because they seal the canal system, stop microleakage and trap any remaining microorganisms. However, because sealers can contact periapical tissues through the apical foramen or lateral canals, their chemical composition and how well they integrate with the body may affect pain and healing after surgery [7, 8-9]. There are many different kinds of sealers used in endodontic practice today. These include calcium hydroxide-, resin-, bioceramic- and bioactive glass-based sealers. These materials have different physical and chemical properties, antimicrobial activity and tissue compatibility, which could affect the body's inflammatory response after surgery [10, 11- 12]. Therefore, it is of interest to compare post-operative pain related to various root canal sealers in single-rooted premolar teeth exhibiting symptomatic apical periodontitis.

## Methodology:

## Study design:

This study was conducted as an in vivo randomized clinical trial to assess post-operative pain associated with various root canal sealers. The Institutional Ethics Committee of People's College of Dental Sciences and Research Centre in Bhopal (Ref No: PCDS/IEC/2024/4/219) gave its approval for the study.

## Study population:

The study included 135 systemically healthy patients aged 18-45 years who presented to the Department of Conservative Dentistry and Endodontics with symptomatic apical periodontitis in single-rooted premolar teeth. The sample size was evenly split into five groups, with each group having 27 people. The groups were based on the type of root canal sealer used.

## Eligibility criteria:

Patients with single-rooted premolars diagnosed with symptomatic apical periodontitis and devoid of systemic illness were included. Patients were excluded if they exhibited root resorption, immature dentition, calcified canals, significant crown destruction, prior root canal therapy, severe malocclusion or a periapical index score exceeding 2. People who had taken painkillers or anti-inflammatory drugs in the last four hours were also not allowed to participate.

## Randomization and group assignment:

Block randomization was used to ensure that participants were evenly split into five groups.

The groups were divided based on

The type of sealer used during obturation:

[1] Group I: Sealer made with zinc oxide eugenol

[2] Group II: Sealer made with calcium hydroxide

[3] Group III: Sealer made of resin

[4] Group IV: Sealer made of bioceramics

[5] Group V: Sealer made of bioactive glass.

## Data collection:

To reduce operator bias, only one skilled operator performed all procedures. After giving the patient local anesthesia and putting a rubber dam around the area, an access cavity was made and pulp tissue was removed. An electronic apex locator and a size 10 K-file were used to find the working length. Root canal instrumentation was performed with pro-Taper rotary nickel-titanium files up to size F3. During instrumentation, a 30-gauge side-vented needle was used to irrigate the canals with 3% sodium hypochlorite. The needle was placed 1 mm short of the working length. After cleaning and shaping, sterile paper points were used to dry the canals and then gutta-percha and the assigned root canal sealer were used to fill them using the single-cone technique. To make sure the obturation was done well, we took X- rays after the surgery.

## Outcome assessment:

The Visual Analog Scale (VAS), ranging from 0 (no pain) to 10 (worst pain imaginable), was used to measure pain after the procedure. Pain scores were recorded at 6, 12, 24 and 48 hours after treatment, as well as on days 3, 5 and 7. According to VAS scores, pain levels were divided into four groups: no pain (0), mild pain (1-3), moderate pain (4-6) and severe pain (7-10).

## Data analysis:

All gathered data were input into SPSS statistical software for examination. We used analysis of variance (ANOVA) to compare mean VAS scores between groups and then performed post hoc tests when needed. A p-value of less than 0.05 was deemed statistically significant.

## Results:

The current randomized clinical trial assessed post-operative pain after root canal obturation employing five distinct root canal sealers. At the initial post-operative time points of 6 and 12 hours, statistically significant differences in pain levels were observed among the groups. The bioactive glass-based sealer consistently had the lowest average VAS scores, which means that it caused the least pain after surgery. The zinc oxide eugenol-based sealer, on the other hand, had the highest pain scores in the first few days after surgery, indicating greater initial tissue irritation. Bioceramic sealers resulted in lower pain scores than calcium hydroxide and resin- based sealers, with the latter two exhibiting similar post-operative pain. The intensity of post-operative pain significantly diminished in all groups at 24 and 48 hours (Table 1 - see PDF). Even though statistically significant differences remained at these times, the overall pain levels were mild and decreased over time. The bioactive glass-based sealer consistently exhibited the lowest pain scores, while the other sealers displayed comparable outcomes with only slight discrepancies. These results indicate that while the type of sealer may affect early post-operative pain, the disparities generally diminish over time as healing occurs.

## Discussion:

After a root canal pain is a common concern that can affect a patient's comfort and how successful they feel the treatment was. In this study, the type of root canal sealer significantly affected early post-operative pain levels. The results indicated that bioactive glass- based sealers yielded the least post-operative pain, while zinc oxide eugenol-based sealers exhibited the highest pain scores in the early post-operative period (6-12 hours). Pain levels in all groups gradually decreased over time and after 48 hours, they were almost gone. Several clinical studies assessing obturation materials and techniques have documented analogous trends of diminishing post-operative pain following endodontic treatment [13, 14-15] the lower pain scores observed with bioactive glass sealers may be due to their high biocompatibility and bioactivity. Bioactive glass releases calcium and phosphate ions that promote hydroxyapatite formation and periapical tissue healing. These reactions help reduce inflammatory responses in the periapical tissues, which might explain why this group experienced less pain [16, 17-18] previous research has indicated that bioactive materials exhibit beneficial tissue responses and may facilitate expedited periapical healing [19, 20, 21, 22- 23]. Bio ceramic sealers exhibited comparatively low pain levels in the current study. These materials are known to have a high pH, good sealing ability and bioactive properties that help with dentin mineralization and fight bacteria. Numerous clinical studies have indicated that calcium silicate-based sealers yield post-operative pain levels that are similar to or marginally lower than those associated with conventional sealers [24, 25] this study found that the zinc oxide eugenol sealer caused the most pain in the first few days after surgery. Eugenol has been documented to induce mild cytotoxic reactions when extruded beyond the apex, potentially resulting in transient inflammation and discomfort in adjacent tissues [26, 27-28]. Clinical trials comparing traditional ZOE sealers with newer bioactive sealers have yielded analogous results [29, 30] another significant finding in this study was the gradual decrease in pain over time among all groups. This result aligns with prior studies indicating that post-operative pain typically peaks within the first 24 hours and progressively diminishes as inflammation subsides and healing begins [31, 32]. The results of this study indicate that post-operative pain is affected by various factors and the biological characteristics of root canal sealers may contribute to alleviating early post-operative discomfort.

## Conclusion:

Bioactive glass-based sealers exhibited the lowest pain scores in the early post-operative phase, indicating superior tissue compatibility and a diminished inflammatory response. Bioceramic sealers also caused relatively little pain, while calcium hydroxide-based and resin-based sealers caused moderate but similar pain after surgery. Data shows that bioactive and bioceramic sealers may be beneficial in alleviating early post-operative pain, although all sealers yielded satisfactory clinical outcomes, with symptoms gradually easing.
